# Note on hydrostatic skeletons: muscles operating within a pressurized environment

**DOI:** 10.1242/bio.060318

**Published:** 2024-07-02

**Authors:** Reinhard Blickhan, Tobias Siebert

**Affiliations:** ^1^ Science of Motion, Friedrich-Schiller-University, 07749 Jena, Germany; ^2^ Institute of Sport and Motion Science, University of Stuttgart, Allmandring 28, D-70569 Stuttgart, Germany

**Keywords:** Hydrostatic skeletons, Muscles, Pressure, Spider, Annelid

## Abstract

Muscles and muscle fibers are volume-constant constructs that deform when contracted and develop internal pressures. However, muscles embedded in hydrostatic skeletons are also exposed to external pressures generated by their activity. For two examples, the pressure generation in spiders and in annelids, we used simplified biomechanical models to demonstrate that high intracellular pressures diminishing the resulting tensile stress of the muscle fibers are avoided in the hydrostatic skeleton. The findings are relevant for a better understanding of the design and functionality of biological hydrostatic skeletons.

## Teaser

**Summary:** In biological hydrostats the muscles are affected by the pressure they generate. Adverse internal muscle pressures are avoided by design in annelids, in spiders they are only observed while struggling.

## INTRODUCTION

Muscle cells can be considered as hydraulic units with a constant volume (muscle: Baskin and Paolini, 1967; Böl et al., 2013; muscle fiber: April et al., 1971; Millman, 1998). With a maximal isometric stress of about 200 *kPa* (Josephson, 1993), muscles interact with the pressure of the environment and with external forces induced by internal or external structures or surrounding tissues. A pressure experienced by its membrane is evenly distributed within the cell and the environmental pressure is transmitted to the cell. Muscle stress, i.e. tensile stress generated via myofibrils, does operate on top of this pressure allowing movement in a pressurized environment.

Hydrostatic skeletons are able to generate high pressure differences between compartments. They consist of connective tissue, combined with musculature or of rigid skeletons in combination with musculature (e.g. Chapman, 1975; Kier, 2012). Hydrostatic skeletons form the basic skeletal structure for many invertebrates such as in annelids, and in the tentacles of squid. They are also observed in vertebrates e.g. in the elephant's trunk, in tongues, and in our digestive system (Van Leeuwen, 1997; Klemm et al., 2023; Longren et al., 2023). Within a hydrostatic skeleton, muscular stress is transformed into hydrostatic pressure. This is also valid for the hydrostatic skeleton in arthropods. The pressure is used to stabilize the structure, to generate motion, to transport nutrients and fluids. The basis for pressure magnitude is muscular stress. Pressure magnitude also depends on architecture, i.e. on the arrangement of muscles fibers within the hydrostatic skeleton.

The influence of internal pressure on muscle performance in dependence on design is investigated in several studies (Heukelom et al., 1979; Rabbany et al., 1994; Daggfeldt, 2006; Sleboda and Roberts, 2020). In studies based on Finite-Element-Analysis (Johansson et al., 2000; Oomens et al., 2003; Liang et al., 2006; Böl et al., 2011; Ryan et al., 2020) this is considered in the established constitutional laws. The central hypothesis of this note is that within hydrostatic skeletons the pressure generated is fed back to the muscle fibers and antagonizes the muscle stress within the muscle fibers. This diminishes the performance of hydrostatic skeletons. To our knowledge, there has been no previous investigation of the influence of internal pressure on the performance and design of hydrostatic skeletons.

We introduce an “effective” muscle stress, *σ*=*σ*_*m*_−*P*, quantifying the antagonizing effect of pressure, *P*, within the muscle fiber with respect to the tensile stress, *σ*_*m*_, generated by the fiber without pressure. Using simple modelling combined with data available in literature we illustrate the effect on the performance of muscle skeletal hydrostats in two examples, (1) the piston like pressure generation by longitudinal or pennate muscles driving a piston within a cylinder (spider), and (2) the pressure generated by a circular muscle ring (annelid).

### Models

Internal pressure reduces the maximum pressure to be generated by hydrostatic skeletons. For simplicity and transparency, we ignore compliance of the hydrostats and do not consider changes of shape. Infinitesimal volume changes necessary to generate pressure are ignored. The muscles are assumed to operate isometrically.

#### Transverse systems (spider)

We consider systems in which the muscle fibers cross the cavity under pressure.

**Fig. 1.**
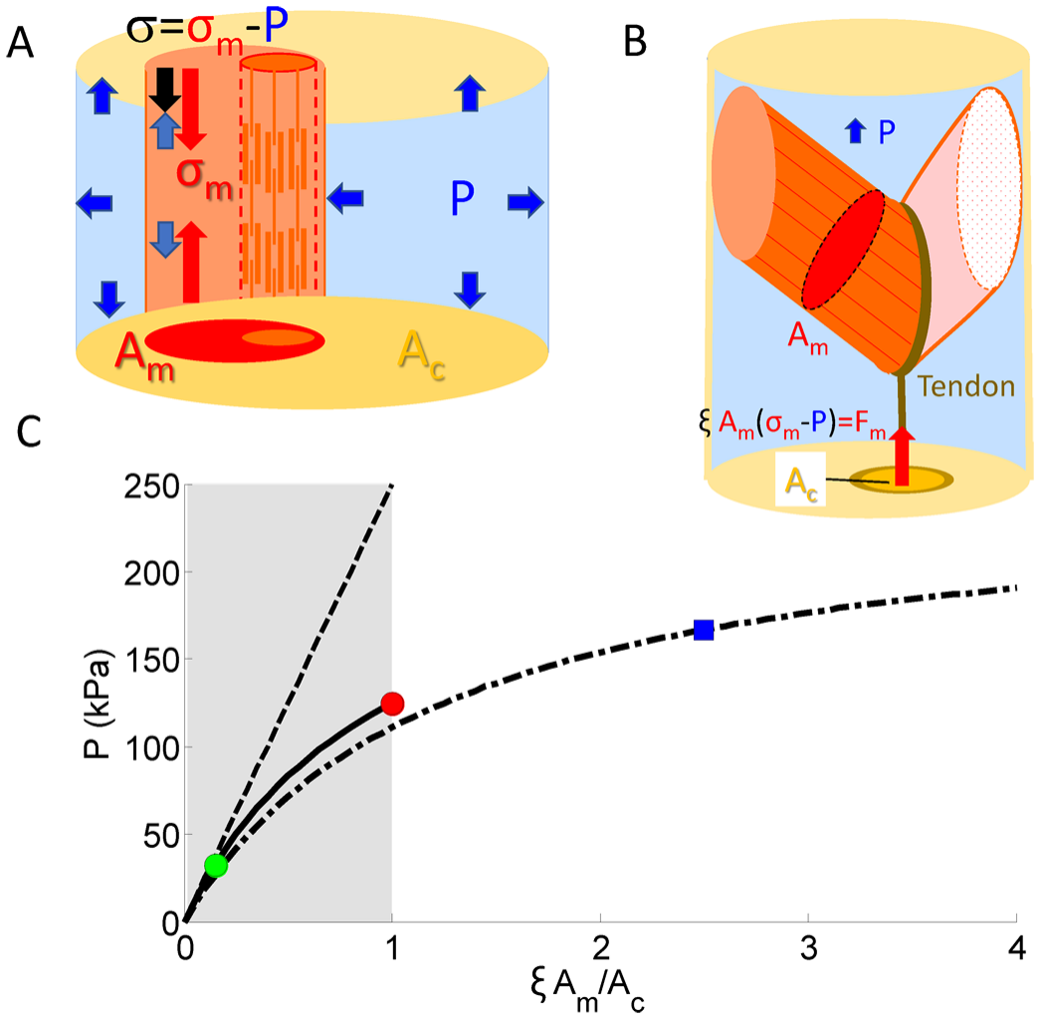
**Cylinder pressurized by longitudinal muscle fibers and a pinnate muscle. Schemes and diagram.** (A) Longitudinal muscle fibers. The muscles (cross-section *A*_*m*_, red; pinnation angle factor *ξ*=1) traverse a fluid filled volume with stiff top and bottom (areas *A*_*c*_) and a stiff side. The stress generated by the muscle filaments, *σ*_*m*_, is counteracted by the pressure *P* generated within the hydrostatic skeleton resulting in a stress of *σ*. (B) Pinnate muscle within a rigid cylinder with a small piston area (*A*_*c*_). The physiological cross-section of muscles perpendicular to the muscle fibers (*A*_*m*_) can by far exceed the piston area. The force transmitted to the piston (*F*_*m*_) via the tendon is reduced due the pinnation angle by a pinnation angle factor *ξ*. (C) Pressure, *P*, in dependence of area ratio, 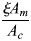. Dashed line: with longitudinal muscle fibers and without stress reduction; solid line: with stress reduction and longitudinal fibers (*ξ*=1); dash-dotted line: pinnate muscle, *ξ*=0.9 for tibia of *Cupiennius salei* (Fig. 2). *A*_*m*_: physiological cross-section of muscle, *A*_*c*_: area of piston; *σ*_*m*_=250 *kPa*. Pressures predicted for *Cupiennius* (see Discussion) - magenta circle: *P*=32.6 *kPa*, activity of *M. ter-end*; red circle: *P*=125 *kPa*, combined action of all muscles attached to the tergum (Fig. 2A,B). Recorded maximum pressure: 65 *kPa* (not depicted; Stewart and Martin, 1974, prosoma, *Dugesiella hentzi*; Anderson and Prestwich, 1975, leg, *Filistata hibernalis*). Blue square: 173 *kPa*, predicted for contraction of all flexors in the tibia. Maximum measured value 130 *kPa* (not depicted). Shaded: range for longitudinal fibers.

Following Blickhan et al. (2021) we assume a cylindrical closed compartment with solid ends (areas: *A*_*c*_; Fig. 1A) filled with fluid (e.g. hemolymph). The volume should be crossed by muscles with cross-section *A*_*m*_ generating a pressure, *P*, relative to the external pressure within the compartment. The muscles generate a tensile stress, *σ*_*m*_. As the muscle fibers are immerged in the pressurized fluid, the pressure is transmitted to the muscle and the resulting fiber stress, *σ*, is reduced, i.e. *σ*=*σ*_*m*_−*P*. To repeat: within each muscle cell, the sum of the forces generated by the myofilaments divided by the cross-sectional area of the cell is diminished by the cells pressure. Similar to the tension of the filaments this pressure is counteracted and transmitted from sarcomere to sarcomere and from cell to cell to the attachment end of the muscle fiber. Correspondingly, the tension generated by the myofilaments, the myofibrils, and the muscle cells is reduced by the internal pressure (Fig. S1B). In a parallel arrangement of muscle fibers, the magnitude of the pressure depends on the relationship between muscle cross-section and the area of the cylindrical compartment: 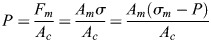. This results in
(1)

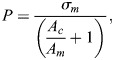
with

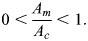


**Fig. 2.**
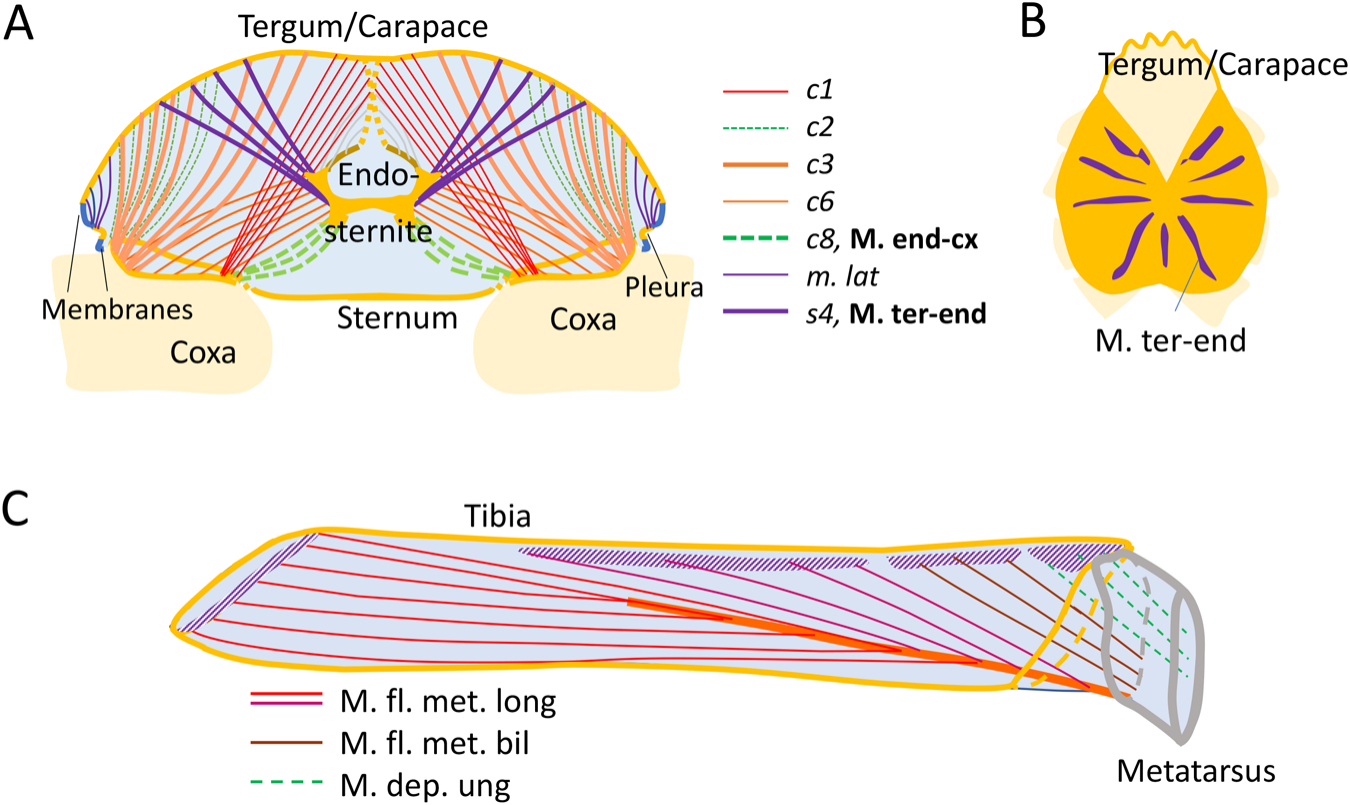
**Scheme of the pressure pump in the prosoma and the leg of the araneid *Cupiennius salei*.** (A) Transversal section (scheme at about the second segment). Pressure is generated by pulling the stiff carapace against the endosternite, the coxae, and the pleura. The endosternite in turn is pulled against the coxae. Legend: Italics (after Palmgren, 1980); normal, bold (after Runge and Wirkner, 2019). (B) Dorsal attachment sites of the *M. ter-end* (magenta) at the tergum. The area shown in gold is completely filled with attachments of muscles running to the endosternite and the coxae (comp. Fig. 2A). (C) Scheme of the tibia and the tibia-metatarsus joint of *Cupiennius salei*. The muscles attach proximally to an apodeme and dorsal attachment sites. Distally they converge to a tendon (*M. fl.met.long*), which attaches to the ventral metatarsus. The *M. fl.met.bil* attaches to the proximal rim of the metatarsus. Roughly, the depicted posterior muscles are mirrored anteriorly. (A,B after Runge and Wirkner, 2019, Eckweiler and Seyfarth, 1988, Palmgren, 1980; C after Blickhan and Barth, 1985).

The maximum pressure with whole compartment area, *A*_*c*_, filled with muscles is *σ*_*m*_/2; for a typical spider (*σ*_*m*_=250 *kPa*; Siebert et al., 2010) results in *P*=125 *kPa* (Blickhan et al., 2021). For 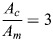, i.e. a cross-section filled with 1/3 muscles this value drops to a quarter of muscle stress (*σ*_*m*_/4). The pressure reduction increases with 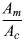 (Fig. 1C). For the spider as an example see Fig. 2 and discussion.

The physiological cross-section can be increased by muscle pinnation and by transfer of muscle force to a tendon (Fig. 1B). Then the physiological cross-section, *A*_*m*_, is not limited any more by the cross-section of the piston, *A*_*c*_, and the ratio 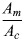 can exceed 1. On the other hand, due to the pinnation angle of the fibers the force is reduced with respect to the physiological cross-section (*F*_*m*_=*ξ σ_m_ A*_*m*_; pinnation angle factor: *ξ*; Fig. 1B). The pressure reduction largely diminishes the muscle force gained by the increased cross-section. (For the theoretical limit of *A*_*m*_=∞ the pressure approaches muscle stress without stress reduction.)

#### Circumferential systems (annelids)

**Fig. 3.**
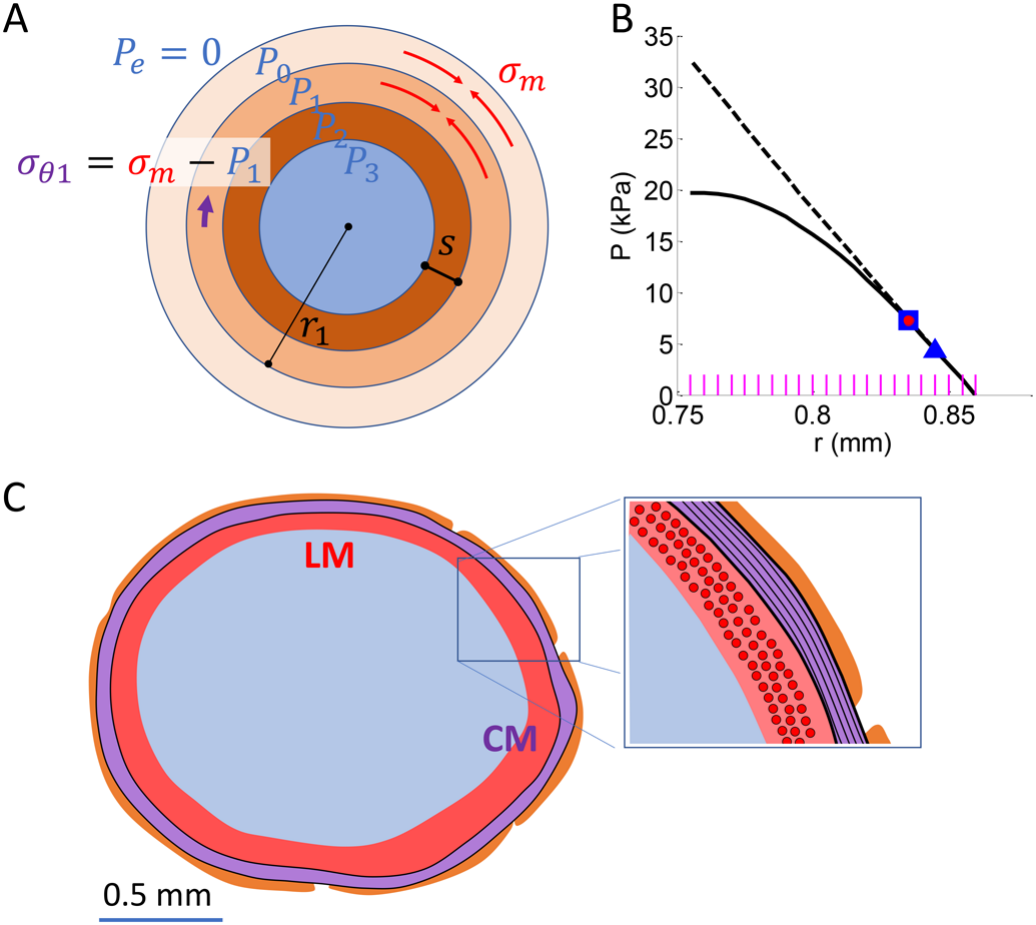
**Cylinder pressurized by circular muscle fibers.** (A) Scheme: pressure increase within a circular muscle structure. *σ*_*m*_: muscle stress; *σ*_*θ*_= tangential stress; *P*_*e*_: external pressure; *P*_1,2,3_: pressure inside the numbered layers; *r*_1_: inner radius of the first layer; *s*: wall thickness. (B) Pressure *P* in dependence of radius *r*. Dashed line: without reducing stress by internal pressure; solid line: assuming reduction by transferred pressure. Markers at abscissa: assumed layers. The maximum, *P*_*max*_=20 *kPa*, is observed after 20 layers. As in *Lumbricus*: starting radius: *r*_*e*0_=0.86 *mm*; layer thickness: *s*=5 μ*m*; muscle stress: *σ*_*m*_=250 *kPa*. Red dot: internal pressure, *P*=7.2 *kPa*, predicted from five circular muscle layers observed in *Lumbricus* (after Kurth and Kier, 2014); blue square: *P*=7.3 *kPa*, recorded pressure during “violent squirming movements” (Seymour, 1969); blue trangle: *P*=4.3 *kPa*, recorded pressure during “violent whole body contraction” (Quillin, 1998). Calculations with Matlab R14 (Mathworks, Natick, MA, USA). (C) Areas of circular (CM, purple) and longitudinal (LM, red) muscle layers in a transverse section of *Lumbricus terrestris* (after Kurth and Kier, 2014).

We consider a circumferential muscle arrangement using the simplest approximation (Fig. 3A; Fig. S1).

The difference between internal (*P*_*i*nt_) and outer (*P*_e_) pressure transmitted by a thin walled tube under tangential stress (*σ*_*θ*_) is (‘Kesselformel’; e.g. Chapman, 1975)
(2)

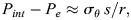
with *s*: wall thickness; *r*: radius. Within a muscle fiber, stress and pressure are constant. With the radius falling layer by layer, the pressure is increasing. The inner muscle fiber layer, layer number *i*, experiences the pressure generated from the outer layers, layer number (*i*−1). This pressure antagonizes muscle stress, i.e. *σ*_*θi*_=*σ*_*m*_−*P*_*i*_. This results in a rapid drop of the generated pressure from layer to layer (Fig. 3B) and makes a high number of layers inefficient. The drop is sensitive to layer thickness, for example, as defined by the diameter of the muscle fibers.

## DISCUSSION

The examples illuminate how the stress reduction by the pressurized environment as calculated above affects the design and operation of biological hydrostatic skeletons.

### Spiders

Measured pressures are within the window predicted for a cylinder pressurized by internal longitudinal muscles.

In spiders, the prosoma is the pump providing the pressure to extend major leg joints (Parry and Brown, 1959; Stewart and Martin, 1974; Runge and Wirkner, 2019). Currently, it is conceived that the muscles running from the tergum or carapace to the endosternite (*M. ter-end*; nomenclature: Runge and Wirkner, 2019) supported by the muscles running from the endosternite to the medial coxa (*M. end-cx*; Fig. 2A) are dedicated to generating prosomal pressure. The complete carapace represents the attachment site of muscles running more or less dorso-ventrally to the endosternite, the pleural sternites embedded in the pleural membrane, and the coxae (Runge and Wirkner, 2019). The dorsal attachment sites of the *M. end-cx* to the endosternite occupy only about 13% of the carapace area (excluding the frontal segment with the mouth part; *Cupennius salei;* Fig. 2B). Following anatomical drawings (Eckweiler and Seyfarth, 1988; Palmgren, 1980) and µCT-scans (Runge and Wirkner, 2019), the muscles are parallel fibred and run to their respective attachment sites. All muscles attached to the carapace affect hemolymph pressure by contraction. Taking the attachment areas (Fig. 2B) as a measure for physiological cross-section, this leads to a predicted peak pressure range from 32.6 *kPa* (*M. ter-end*; *M. end-cx*) to maximal 125 *kPa* (all muscles or complete carapace Fig. 2B; *Cupiennius salei*). If we consider an average fiber angle of 45° with respect to the dorsad working axis of the pressure pump (Fig. 2A) in the calculation the predicted maximum pressure drops to 100 *kPa*. The recorded maximum pressure was 65 *kPa* (Stewart and Martin, 1974, prosoma, *Dugesiella hentzi*; Anderson and Prestwich, 1975, leg, *Filistata hibernalis*). About half of the muscles attached to the carapace (*Am/Ac*=0.5) including those driving the coxae seem to be involved in generating these maximum pressure values.

The pressure in the legs especially at its major joints can be generated by flexion. Here, the tube of the leg segment offers the possibility to increase the areas for muscle attachments. We take the tibia and the tibia-metatarsus joint as an example (Fig. 2C). The hemolymph volume displaced is located in the joint area. A muscle, *M. fl.met.long*, attaching to the proximal apodeme at the tibia and at the dorsal tibia, converges to a tendon which in turn is attached ventrally to the metatarsus. The *M. fl.met.bil* attaches proximally at the dorsal tibia and distally to the proximal rim of the adjacent leg segment. The physiological cross-sectional area, the area perpendicular to the muscle fibers, sums up to about 1.5% of the cross-sectional area of the joint. The shallow fiber angles diminish this physiological cross-section by less than 10%. At the joint the different advantage of the muscles and of the pressure area must be considered. This leverage leads to an increase of the effective area ratio by a factor of two at maximum and would result in a maximum pressure estimate of 173 *kPa* in the spider's tibia (Fig. 1C). The highest values recorded in the tibia during autonomy was 130 *kPa* (Blickhan and Barth, 1985). This pressure is also close to the value estimated for the maximum prosomal pressure (see above). A flexing leg and the maximal compression of the prosoma combined may provide the suitable pressure for autonomy. These estimates assume a closed compartment, i.e. no draining of the pressurized fluid into adjacent segments or a proximal counter pressure. This assumption may only hold for very short dynamic contractions and during extreme activity. With an injured leg, open at the femur, the spider may not be able to generate counter pressure. Then, the muscles attaching the sclerites of the trochanter (Parry, 1957) may not be able to autotomize. Simultaneous registration of pressure in prosoma and leg combined with an anatomical estimate of maximum muscle forces would help to consolidate our observations.

### Annelids

The common earthworm (*Lumbricus terrestris*) represents a frequently cited example of a hydrostatic skeleton (Chapman, 1975; Kier, 2012). Its peristaltic movement is generated by the activity of circular and longitudinal muscles (Fig. 3) segmentally distributed along its body (Newell, 1950; Chapman, 1975).

Typical internal pressures recorded while moving by peristalsis during the phase of circular muscle activity reach about 700 *Pa* (*Lumbricus terrestris*, Quillin, 1998). The scaling of this pressure with respect to body mass recorded during locomotion is only marginal (Quillin, 1998). However, values measured during “violent whole body contraction” range from 4.3 *kPa* (1.8 *gr* earthworm; Quillin, 1998) to 7.3 *kPa* “during violent squirming movements” (6 *gr Lumbricus terrestris*, Seymour, 1969). This corresponds to the values predicted from the calculation with maximum muscle activity (Fig. 2A; assumed muscle stress 250 *kPa*; muscle cell diameter of 5 *μm*, five layers, radius 0.86 *mm*; *Lumbricus terrestris*, after Kurth and Kier, 2014). Direct measurements of isometric force in the literature were not converted into stress (*Pheretima communissima,* Tashiro, 1971; *Pheretima communissima,* Toida et al., 1975).

Longitudinal muscles generate higher internal pressures during their active phase while moving by peristalsis (*Lumbricus terrestris*; Quillin, 1998). When expansion is hampered, as during burrowing, maximal radial pressure reached 80 *kPa* while passing a hole diameter of 2 *mm* (mass *ca*. 0.5 *gr*; *Aporrectodea caliginosa*, Stovold et al., 2003). By reducing the diameter of the earthworm from an uncompressed 3 *mm* to 2 *mm* the percentage of the longitudinal muscles to the cross-sectional area increases from about 20% to 50%. From this (Equ. 2, *σ*_*m*_=250 kPa) an internal pressure of 83 *kPa* can be predicted.

### Muscle pressure in hydrostats

Fiber curvature and external loads cause pressure within skeletal muscles (Van Leeuwen and Spoor, 1993; Azizi et al., 2017). There too, high pressure values would antagonize force generation. Measurement of inter-muscular and intra-muscular pressure is challenging (Davis et al., 2003; Reinhardt et al., 2016). The pressure values predicted for annelids and spiders (Figs 1C and 3B) are within the pressure range reported in the few available experimental studies. Maximal inter-muscular pressure between bellies of rabbit leg muscles reached 70 *kPa* (Reinhardt et al., 2016). Lateral loads, generating local pressure of 13 *kPa* in the contact area in between muscles, diminish muscle force by 5% (Siebert et al., 2014a,b). Muscle compression with elastic bandages or slings also influences force-generating abilities (Wakeling et al., 2013). Sleboda and Roberts (2020) found that squeezing a frog muscle at short muscle length with a pneumatic cuff at an inflation pressure of 35 *kPa* reduces generated force by about 12% (0.12·250 *kPa*=30 *kPa*). Consequently, experimental studies at the muscle level also indicate an influence on muscle force by external pressure. Intramuscular hydrostatic pressure have been measured under various conditions and for different purposes (examples for maximal pressures: frog *M. gastrocnemius*, 40 *kPa*, Hill, 1948; human *M. supraspinatus*, during maximum voluntary contraction 70 *kPa*, Järvholm et al., 1991; rabbit *M. tibialis anterior*, isometric 3.3 *kPa*, Ward et al., 2007). The measured pressure is influenced especially by fiber curvature and the vicinity of attachment sites. Otten (1988) used a calculation of internal pressure in dependence of muscle fiber curvature. Unfortunately, this study did not consider stress reduction due to pressure within the fiber.

Classic Hill-type muscle models do not take fluid dynamics into account. They calculate forces based on phenomenological dependencies such as the force-length curve and the force-velocity relationships (Siebert et al., 2008; Haeufle et al., 2014). Extended Hill-type muscle models (Siebert et al., 2018) that consider an interaction with transverse forces can describe the qualitative influence of external pressures on muscle strength. A series of theoretical muscle models predict that intramuscular pressure should influence muscle performance by directly opposing sarcomere shortening forces (Heukelom et al., 1979; Rabbany et al., 1994; Daggfeldt, 2006; Sleboda and Roberts, 2020). More recently, detailed 3D finite element models (FEM) considered the internal pressure overlaying and in effect reducing muscle stress (Johansson et al., 2000; Oomens et al., 2003; Liang et al., 2006; Böl et al., 2011; Ryan et al., 2020). The models, however, do not consider the size of the biological hydrostatic units (cells). Our circumferential model is sensitive to layer thickness. Pressure is constant within a layer. For the annelids we assume the thickness as being defined by the diameter of a muscle fiber. In larger hydrostats, layer thickness might be defined by connective tissue. In standard FEM-models the element, i.e. the special resolution of the meshwork, is defined by computational boundaries. Jenkyn et al. (2002), documented an agreement between the FEM-model and direct measurements (Davis et al., 2003) in the center of the unipennate rabbit *M. tibialis anterior* considering basic muscle properties (2.7 *kPa* at resting length). In the squid tentacle, short term high pressures of 20 *kPa* are essential for the acceleration of (Van Leeuwen and Kier, 1997). In general, high pressures are avoided in order to enhance mechanical muscle efficiency. In order to evaluate the influence of internal pressure on the muscle design, architectures with curvatures and thicknesses exceeding biological examples should be studied.

### Pressure antagonizes muscle tension

We did not perform our own pressure measurements. We consider it supportive of our modelling approach that the limited data available in the literature coincide with our predictions. The fact that the pressure measured in the hydrostats does not reach values which could be calculated ignoring the antagonizing pressure within the muscle cells could be due to muscle recruitment in case of the spider and due to neglected compliance in the case of the annelid. More measurements and more elaborate modelling will be necessary to support our results. However, also in models considering deformation and connective tissue the physical fact that the pressure generated feeds back to the pressure within the muscle fibers should lead to substantial modifications of the predictions.

In systems submerged in a pressurized environment as in fish the pressure within the muscles corresponds to the pressure in the environment with minor deviations (e.g. induced by the curvature of muscle fibers; Fig. S1A). The muscles pressurizing the prosoma of spiders are submerged in the pressurized fluid, with the exception of their attachment site where muscle tension is transmitted to the carapace and the environment. The pressure pushes against the carapace and the muscle fibers pull at the carapace (Fig. 1; Fig. S1B). The muscle stress is partially compensated by the internal pressure. In our model we consider this by introducing a reduced “effective” muscle stress.

In the circular arrangement as in skeletal muscles the curvature of the fiber generates a pressure perpendicular to the fiber in the direction of the radius of curvature. As there is no pressure gradient within each muscle fiber, there is a stepwise increase of pressure from layer to layer (Fig. 2; Fig. S1C). Presumably, connective tissue helps to organize the layered structure and to maintain the pressure gradient within the wall of the annelid. In our approach, due to the ring-like arrangement stress generated in the tangential direction by the muscle fiber is assumed to be antagonized by the adjacent muscle fiber within the ring. Similarly, the pressure generated by the surrounding circular layer is assumed to be distributed in the muscle fiber. At any radial cut the tangential pressure is in this situation antagonized by a corresponding opposite pressure. The pressure antagonizes the tension generated by the muscle fibers. Consideration of connective tissue and form changes might lead to modifications. However, we predict that the transmission of the pressure into the muscle cells will still affect the magnitude of the generated pressure and the suitable design of the muscular hydrostat.

### Conclusion

Pressure transmitted to the muscle cells partly compensates the tensile stress generated by the muscle fibers. This in turn reduces their ability to produce pressure within hydrostatic skeletons. Strong reductions in muscle stress are avoided by design or only accepted during short activities under maximum effort. In the spider's transverse systems, this is achieved by limiting the fibers (cross-section) recruited for pressure generation during locomotion. Within slender skeletons (legs) with pressurized pistons of small diameter (joints), designs with higher muscle volumes are possible to enhance pressure generation. In hydrostatic skeletons, in which a circular arrangement of muscle fibers is used to generate pressures as in annelids, the appropriate number of muscle layers and thus suitable wall thickness is predicted to be limited. We conclude from our studies that with increasing wall thickness the ability of circular hydrostats to generate pressure is diminished. The resulting design criteria influence the phylogenetic development of skeletal hydrostats widely distributed in nature.

## Supplementary Material

10.1242/biolopen.060318_sup1 Supplementary information
