# Supplementary material for: Note on hydrostatic skeletons: muscles operating within a pressurized environment
Source: Biol Open. 2024 Jul 2;13(7):bio060318. doi: 10.1242/bio.060318 (PMC11261639; doi:10.1242/bio.060318)
Supplement: Supplementary information [file biolopen-13-060318-s1.pdf]

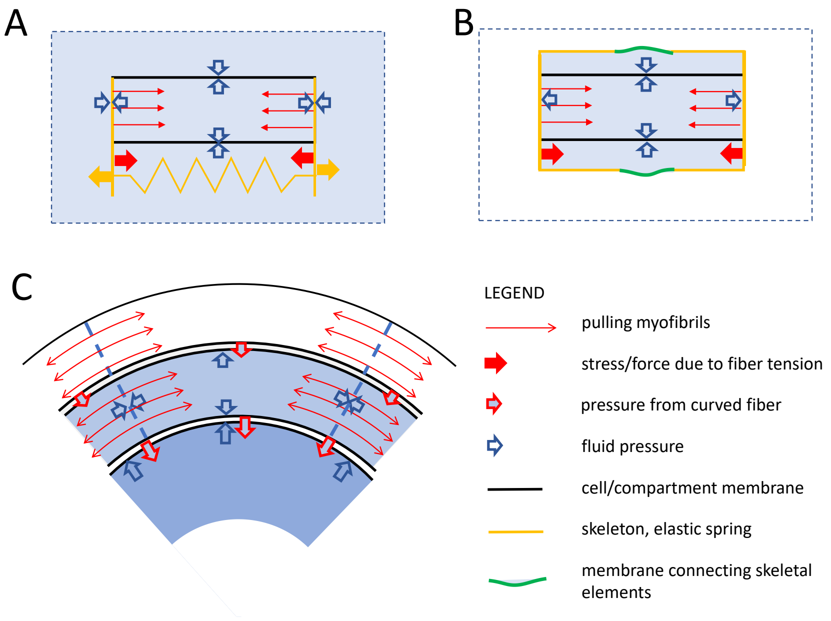

Fig. S1. Muscle fibers under pressure. A) Muscles submerged in a pressurized environment (e.g. fish). With minor deviations (curvature etc.) the pressure within the muscles corresponds to the pressure in the environment. Tensile stress generated by the muscle and working against internal or external load (spring) is not diminished by the pressure. B) Muscle-skeletal hydrostat (e.g. spider). The pressure generated by compressing skeletal segments differs from the environmental pressure. The pressure inside the cavity is transmitted to the muscle fiber and diminishes the resulting stress. C) Muscle fibers in circular hydrostats (e.g. annelid). Proportional to its curvature, the muscle fiber generates pressure on its concave side (red unfilled arrows). The next inner fiber layer is exposed to this pressure. This pressure antagonizes fiber stress and reduces the generated pressure. With the support of connective tissue compartments with constant pressure may consist of several layers of muscle fibers.

Biology Open • Supplementary information

Matlab function to calculate pressure generated by a circular muscle ring.

```
function [r,p1,p2,ntm]= PressureCircMus(sigm,s,ro,nt)
% Increase of pressure from outside to the next layer.
% With constant muscle tension but reduced by increasing pressure.
% (Data from Lumbricus)
% Input:
%   sigm: muscle stress; sigm = 250E3Pa
%   s:    layer thickness;  s = 5E-6 m
%   ro:   radius of outer layer; ro = 8.6E-4 m
%   nt:   maximum number of iterations assumed (25)
%
% Output:
%   r:   vector with raddii
%   p1:  pressure values without stress reduction
%   p2:  pressure values with reduction
%   ntm: maximum number of iterations performed
%
% %%%%%%%%%%%%%%
% R. Blickhan  %
% 20.09.2023   %
% %%%%%%%%%%%%%%%%%%%%%%%%%%%%%%%%%%%%%%%%%%%%%%%%%%%%%%%%%%%%%%%%%%
sigmi(1:nt) = NaN; r(1:nt)      = NaN; p1(1:nt)     = NaN; p2(1:nt)     = NaN;

sigmi(1)=sigm; % starting values for iteration
i = 1;
r(1)= ro;
p1(1) = 0;
p2(1) = 0;

% Iteration
while sigmi(i)>0              % until pressure compensates fiber tension
    sigmi(i+1) = sigmi(i)-p2(i);    % iteratively reduced tension
    r(i+1) = ro-i*s;                % incremental reduction of radius
    p1(i+1)=sigm*s/r(i+1)+p1(i);    % 'Kesselformel'
    p2(i+1)=sigmi(i+1)*s/r(i+1)+p2(i); % including stress reduction
    i = i+1;
end
ntm = i; % number of iterations until sigmi(i)<=0
```

Biology Open • Supplementary information
